# Solid Pseudopapillary Neoplasm of the Pancreatic Body and Tail in a 14-Year-Old Female Patient: A Report of a Rare Case and Literature Review

**DOI:** 10.7759/cureus.96675

**Published:** 2025-11-12

**Authors:** Yunuén Maqueda Sánchez, Luis E Fregoso Arteaga, Marcos Pares Alvarado, María E Tenorio Lázaro, Abraham Cañavera Constantino, Romario Sánchez Villalobos, Erick P Ramírez Gómez, Violeta Ibarra Silverio, Guadalupe M Santana Salas, Gloria Barrera Sánchez, Oliver B Harris Guzmán

**Affiliations:** 1 General Surgery, Hospital General de Cancún “Jesús Kumate Rodríguez”, Universidad Autónoma de Yucatan (UADY), Cancun, MEX; 2 Oncologic Surgery, Centro Estatal Oncología Campeche, Campeche, MEX; 3 General Surgery, Hospital Regional de Alta Especialidad de la Península de Yucatán (HRAEPY), Mérida, MEX; 4 Radiation Oncology, Centro Estatal Oncología Campeche, Campeche, MEX; 5 Pathology, Centro Estatal Oncología Campeche, Campeche, MEX; 6 General Medicine, Universidad Autónoma de Nayarit, Tepic, MEX; 7 Anesthesiology, Hospital General Zacatecas "Luz González Cosio", Zacatecas, MEX; 8 General Surgery, Hospital General de Cancún “Jesús Kumate Rodríguez”, Universidad Autónoma de Yucatan (UADY), Cancún, MEX; 9 General Surgery, Instituto de Seguridad y Servicios Sociales de los Trabajadores del Estado (ISSSTE) Tláhuac "Dra. Matilde Petra Montoya Lafragua", Mexico City, MEX

**Keywords:** body and tail pancreas, case report, distal pancreatectomy, frantz tumor, pancreatic tumor, pediatric pancreas tumor, pediatric surgery, rare pancreatic tumor, solid pseudopapillary neoplasm, spleen preservation

## Abstract

Solid pseudopapillary neoplasms (SPNs) of the pancreas are rare tumors. They typically affect young women and have low malignant potential. We describe the case of a 14-year-old female patient who presented with an abdominal mass diagnosed as a SPN of the pancreatic body and tail. The patient underwent a spleen-preserving distal pancreatectomy, and histopathology with immunohistochemistry confirmed the diagnosis. Her postoperative course was uneventful. This report underscores the importance of considering SPNs in the differential diagnosis of pediatric pancreatic tumors and highlights the favorable prognosis associated with complete surgical resection.

## Introduction

Solid pseudopapillary neoplasms (SPNs) of the pancreas are rare low-grade malignant tumors that account for approximately 1-3% of exocrine pancreatic tumors [1]. They predominantly affect young women, especially during the second and third decades of life [2]. Although they are often detected incidentally, patients may present with abdominal pain, a palpable mass, or nonspecific gastrointestinal symptoms [3]. Advances in imaging techniques and minimally invasive surgery have improved diagnostic accuracy and management of SPNs in both adult and pediatric populations [4,5]. Complete surgical resection remains the treatment of choice, offering excellent long-term outcomes [6,7].

If left untreated, SPNs may continue to enlarge, causing compression of adjacent organs or, rarely, malignant transformation. Early diagnosis and resection are essential to prevent complications.

## Case presentation

A 14-year-old girl presented with a three-month history of intermittent, dull epigastric pain that gradually increased in intensity and frequency. The pain was moderate (rated 6/10), non-radiating, and occasionally interfered with daily activities. It was not associated with meals, nausea, or vomiting. There was no history of jaundice, fever, or trauma. The patient reported mild weight loss of approximately 3 kg over two months.

On physical examination, she appeared well-nourished and in no distress. Vital signs were within normal limits. Abdominal examination revealed mild tenderness in the epigastric region without palpable mass, organomegaly, or peritoneal signs. No lymphadenopathy or other abnormalities were identified on systemic examination.

**Figure 1:**
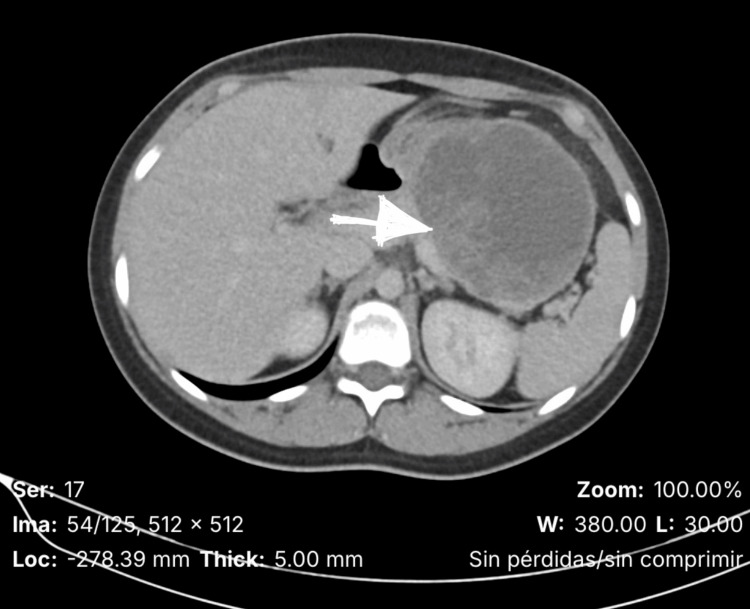
Abdominal CT Scan Showing a Well-Defined Lesion in the Pancreatic Body and Tail (Arrow Indicates the Area of Interest).

Abdominal ultrasound demonstrated a well-circumscribed heterogeneous mass in the pancreatic body, prompting further evaluation with a contrast-enhanced computed tomography (CT) scan. The CT revealed a 6.2 × 5.4 cm encapsulated lesion with mixed solid and cystic components located in the pancreatic body and tail, without vascular invasion or distant metastasis (Figure 1).

Laboratory findings are summarized in Table 1. Results showed leukocytosis (16,100/µL) with neutrophilia (81.8%) and elevated C-reactive protein (26.8 mg/L), while liver and pancreatic enzyme levels were within normal limits.

**Table 1:** Laboratory Findings.

Parameter	Result	Reference Range
White blood cell count	16,100 /µL	4,000–11,000 /µL
Neutrophils	81.80%	40–75%
C-reactive protein	26.8 mg/L	<5 mg/L
Amylase	78 U/L	30–110 U/L
Lipase	52 U/L	13–60 U/L
AST	32 U/L	10–40 U/L
ALT	29 U/L	10–45 U/L
Total bilirubin	0.8 mg/dL	0.1–1.2 mg/dL

**Figure 2:**
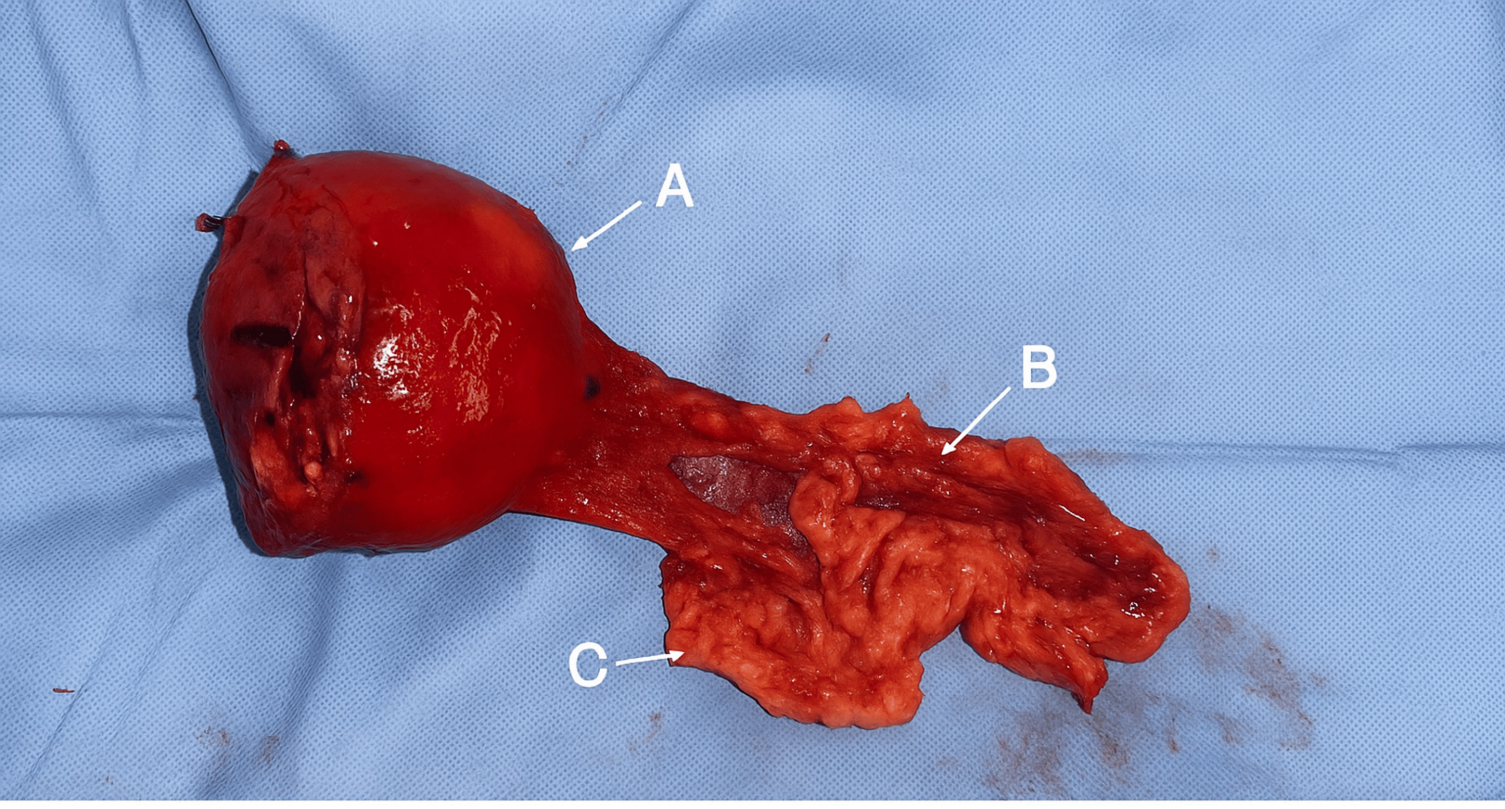
Surgical Specimen Findings. (A) Gross specimen showing a well-encapsulated solid-cystic mass involving the pancreatic body and tail (Frantz tumor). (B) Section of the pancreatic body after distal pancreatectomy with splenic preservation. (C) Attached omental tissue.

The patient underwent a spleen-preserving distal pancreatectomy. Intraoperative findings confirmed a well-encapsulated, friable mass confined to the pancreatic body and tail. Gross examination revealed areas of hemorrhage and necrosis (Figure 2).

**Figure 3:**
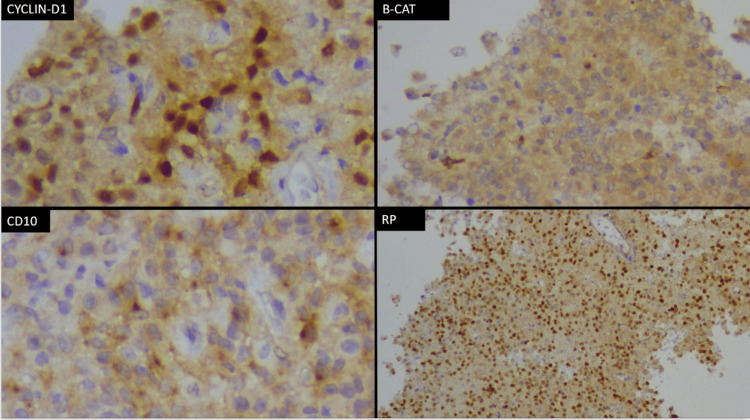
Histopathological Images Showing Pseudopapillary Structures (H&E Stain, ×40). Immunohistochemistry: Upper left: Cyclin-D1; Upper Right: B-cathenin; Lower Left: CD10; Lower Right: Progesterone Receptor.​

Histopathological evaluation demonstrated pseudopapillary structures composed of uniform epithelial cells surrounding fibrovascular cores. Immunohistochemistry was positive for β-catenin (nuclear and cytoplasmic), vimentin, CD10, and progesterone receptor, consistent with an SPN (Figure 3).

The postoperative course was uneventful. The patient tolerated oral intake on postoperative day 3 and was discharged on day 7. At 12-month follow-up, she remained asymptomatic, with complete resolution of abdominal pain and no evidence of recurrence on abdominal ultrasound.

The case was managed by a multidisciplinary team including pediatric surgery, radiology, pathology, and anesthesiology specialists, ensuring comprehensive perioperative care and optimal outcomes.

Histopathological examination revealed pseudopapillary structures with areas of hemorrhagic degeneration, consistent with an SPN (Figure 3). 

Immunohistochemistry supported the diagnosis, with positivity for β-catenin, vimentin, CD10, and progesterone receptor. The detailed immunohistochemical profile is summarized in Table 2.

**Table 2:** Immunohistochemistry.

Antibody	Finding
B-catenin	Focal cytoplasmic and membranous positivity
E-cadherin	Focal cytoplasmic and membranous positivity
Cyclin-D1	Positive nuclear expression
Progesterone receptor	Positive diffuse nuclear expression
CD10	Cytoplasmic and membranous positivity

The postoperative course was uneventful. The patient tolerated oral liquids on the first postoperative day, advanced to a regular diet the same day, and was discharged on postoperative day two in good condition, without pain or fever.

## Discussion

SPNs are rare pancreatic tumors with low malignant potential, representing less than 3% of all pancreatic neoplasms [8]. They predominantly affect young women and typically follow an indolent course.

Early surgical resection is the treatment of choice because complete excision is usually curative and prevents local complications or malignant transformation. Malignant potential has been reported in approximately 10-15% of cases, while recurrence after complete resection is uncommon (<5%) [9]. If left untreated, SPNs may continue to enlarge, compressing nearby organs or, in rare cases, invading surrounding structures [10].

In pediatric patients, early recognition and resection are essential to prevent disease progression and ensure long-term cure. Minimally invasive and spleen-preserving techniques have shown comparable oncologic outcomes with reduced morbidity and faster recovery [11]. Long-term follow-up is recommended, as late recurrence, although rare, has been described even a decade after surgery [12].

This case contributes to the existing literature by providing detailed management and follow-up data in a pediatric patient. It supports the growing evidence that spleen-preserving distal pancreatectomy is a safe and effective approach, maintaining immunologic function while achieving complete tumor removal.

## Conclusions

SPNs of the pancreas, though rare, should be considered in the differential diagnosis of pancreatic masses in pediatric patients. Early recognition and complete surgical resection provide excellent outcomes. Preservation of the spleen, whenever technically feasible, reduces long-term complications. This case underlines the importance of multidisciplinary management and careful postoperative follow-up to ensure optimal recovery in children affected by this tumor.
